# Targeting self-reported and neural error sensitivity: Short- and long-term effects of a one-week online intervention

**DOI:** 10.1016/j.ijchp.2026.100667

**Published:** 2026-01-23

**Authors:** Kai Härpfer, Franziska M. Kausche, Alexandria Meyer, Norman B. Schmidt, Anja Riesel

**Affiliations:** a Department of Clinical Psychology and Neuroscience, University of Hamburg, Hamburg, Hamburg, Germany; b School of Education and Counseling Psychology, Santa Clara University, Santa Clara, CA, United States; c Department of Psychology, Florida State University, Tallahassee, FL, United States

**Keywords:** Anxiety, Error-related negativity, Error positivity, Error sensitivity, Intervention, Obsessive-compulsive disorder

## Abstract

Elevated error-related brain potentials such as the error-related negativity (ERN) and error positivity (Pe) have been discussed as neural markers of error sensitivity and are thought to reflect increased risk for anxiety and obsessive-compulsive disorders. Consequently, targeting error sensitivity with precise interventions has been found a promising avenue of recent mechanism-based research aiming to reduce this risk. In this preregistered, randomized-controlled trial, we tested the efficacy of a one-week, online intervention designed to reduce error sensitivity. A sample of 237 individuals was randomly assigned to either the intervention or a waitlist control group. Participants completed self-report measures of error sensitivity as well as worry, obsessive-compulsive, and depressive symptoms at pre- and post-intervention, and at an eight-week follow-up. Additionally, neural measures (ERN and Pe) were assessed in a subsample of 69 participants before and after the intervention. Intent-to-treat analyses revealed a medium-sized reduction of self-reported error sensitivity and worry symptoms in the intervention group, with effects persisting at follow-up. Moreover, greater baseline severity and higher intervention adherence were associated with larger reductions of self-reported error sensitivity. In the subsample, no evidence was found for an ERN reduction. However, a reduction in the Pe was observed, indicating diminished error significance and decreased allocation of cognitive resources to erroneous actions. These results suggest that the online intervention reduces both self-reported and neural error sensitivity (Pe but not ERN), offering a low‑threshold, easily disseminable approach with promise as an early prevention tool and as an adjunct to established cognitive‑behavioral treatments.

## Introduction

In a complex and ever-changing environment, humans must learn from previous experiences and adapt their behavior to specific tasks and situations. One approach to learning is trial and error. In the brain, correct and incorrect responses to tasks can be identified by event-related potentials (ERPs), specifically the error-related negativity (ERN) and the correct-response negativity (CRN), respectively (Falkenstein et al., 1991; Gehring et al., 1993). The ERN appears as a negatively peaking waveform typically at fronto-central sites, about 50 ms after an error has been committed. It reflects an early error monitoring process and serves as a neural signal that facilitates behavioral adaptation, enhances task performance, and reduces the likelihood of future errors (Botvinick et al., 2001; Falkenstein et al., 1991; Gehring et al., 1993; Holroyd & Coles, 2002; Yeung et al., 2004). Individual differences in the ERN are considered an indicator of individuals’ error reactivity, i.e. error sensitivity (Meyer et al., 2020). The CRN, by contrast, arises within a similar time window and displays a comparable scalp distribution to the ERN, but appears with a smaller amplitude following correct responses (Ford, 1999; Vidal et al., 2003; Vidal et al., 2000).

Furthermore, a more centro-parietal ERP associated with error processing is the error positivity (Pe, Falkenstein et al., 2000). The Pe is observed as a positive deflection following the ERN, typically occurring between 400 and 600 ms after erroneous responses (Endrass et al., 2007; Overbeek et al., 2005). The Pe is thought to reflect error awareness and error significance (Endrass et al., 2005; Endrass et al., 2007; Kirschner et al., 2020; Nieuwenhuis et al., 2001; Overbeek et al., 2005), or more generally, the allocation of cognitive resources to errors, resulting in the updating of error representations, comparable with the stimulus-locked P3 (see Wessel, 2012, for a review). Similar to the ERN and CRN, a positive but smaller deflection following correct responses can also be observed within a comparable time window and scalp distribution as the Pe; this component is often referred to as the Pc.

Serving as indicators of neural error processing, the ERN and Pe have been shown to reflect dissociable cognitive functions (Di Gregorio et al., 2018; Nieuwenhuis et al., 2001; Overbeek et al., 2005). The ERN is an early, highly automatic neural signal that indexes the detection of an individual’s erroneous actions (Botvinick et al., 2001; Falkenstein et al., 1991; Gehring et al., 1993; Holroyd & Coles, 2002; Yeung et al., 2004), whereas the Pe reflects later, more elaborated and evaluative processing of errors (Endrass et al., 2005; Endrass et al., 2007; Kirschner et al., 2020; Nieuwenhuis et al., 2001; Overbeek et al., 2005). Thus, to assess the sensitivity of an individual’s cognitive system to error commission, not only the ERN constitutes a relevant neural marker, as initially proposed (Meyer et al., 2020), but also the Pe should be considered, thereby capturing both early, automatic and later, evaluative stages of error processing.

A substantial amount of evidence indicates that an altered ERN serves as a neural risk marker of several mental health conditions (Pasion & Barbosa, 2019; Riesel et al., 2025). Specifically, enhanced ERN amplitudes have been associated with anxiety (Moser et al., 2013; Saunders & Inzlicht, 2020; Xie et al., 2025) and obsessive-compulsive disorders (OCD; Bellato et al., 2021; Lamothe et al., 2025; Mathews et al., 2012; Riesel, 2019). ERN-alterations have also been shown to predict progression of anxiety symptoms or even the onset of anxiety disorders (Lahat et al., 2014; Meyer et al., 2015; Meyer et al., 2021; Meyer et al., 2018; Riesel et al., 2021). Moreover, unaffected individuals with a relative suffering from an internalizing disorders (Härpfer et al., 2025)—including anxiety disorders (Riesel et al., 2019) and OCD (Carrasco et al., 2013; Riesel et al., 2011; Riesel et al., 2019)—display similarly elevated ERN amplitudes, potentially indicating an increased risk for developing the respective disorder. Fewer studies have investigated potential differences in the Pe component. However, meta-analyses have shown increased Pe amplitudes in individuals with OCD (Bellato et al., 2021), whereas no such differences have been reported for generalized anxiety disorder (Xie et al., 2025). Taken together, there is convincing evidence to assume, that an elevated ERN can serve as a neural risk marker for disorders of the anxiety and obsessive-compulsive spectrum (Härpfer & Riesel, 2026). In contrast, the clinical utility of the Pe for these disorders remains an understudied area, with initial evidence pointing toward increased Pe amplitudes as a potential neural indicator for OCD. Nonetheless, these markers may represent targets to personalize and augment classical treatment approaches of anxiety and OCD.

Although first-line treatments such as cognitive-behavioral therapy (CBT) have been shown to effectively reduce both anxiety (Gorka et al., 2018; Kujawa et al., 2016; Ladouceur et al., 2018) and obsessive-compulsive symptoms (Hajcak et al., 2008; Riesel et al., 2015), they do not appear to normalize the elevated ERN amplitudes associated with these conditions even after 12 to 30 sessions of CBT. More targeted short-term interventions, such as emotional expressive writing paradigms designed to offload individuals’ working memory from distracting worries, have yielded inconsistent results in reducing the ERN (Härpfer et al., 2022; Schroder et al., 2018). Additionally, relapse rates after CBT remain high, with 10–33% of patients experiencing recurrence (Johnco et al. (2025) in press; Levy et al., 2021; Levy et al., 2022; Lorimer et al., 2021). Since neither CBT nor expressive writing were specifically designed to target error sensitivity, this may suggest that an untreated elevated ERN leaves individuals vulnerable to future increases in anxiety. Consistent with this assumption, an elevated ERN has been identified as a prognostic indicator for symptom progression among clinically anxious individuals (Meyer et al., 2021). Given that an elevated ERN (and potentially an elevated Pe) is considered to indicate risk for anxiety disorders and OCD (Härpfer & Riesel, 2026), as well as for symptom progression (Meyer et al., 2021), reducing error sensitivity may not only lower the risk of developing a respective disorder but also decrease the risk of future relapse in already treated individuals.

Therefore, a novel approach has been developed to directly target the psychological constructs underlying the ERN, such as error sensitivity and concern over performance. Single-session, computer-based tutorials addressing various error-related topics, including perfectionism, fear of social repercussions after errors, and overestimation of the negative consequences of errors, have shown distinct effects in adults and children. Meyer et al. (2020) found a decrease in neural error sensitivity (as reflected by ERN amplitudes) in adults, although self-reported error sensitivity was not assessed in that study. In contrast, Meyer et al. (2023), which investigated children, demonstrated a reduction in self-reported error sensitivity without corresponding changes of the ERN. However, it remains unclear whether such neural changes are sustainable, as the pre- and post-assessments in these studies were conducted within a single day. Furthermore, neither study has investigated the effect on the Pe. Building on these important initial findings, we developed an updated version for adults that extends over three consecutive sessions (each approximately 20 minutes), conducted within one week. This version incorporated additional exercises to be completed between sessions, designed to reinforce learning and promote the transfer to everyday life. Ultimately, these enhancements aimed to achieve a long-term reduction of error sensitivity, thereby potentially mitigating the risk of internalizing psychopathology.

To this end, this study evaluated the efficacy of an online intervention aimed at reducing both self-reported and neural error sensitivity in a sample of mainly university students. We employed a randomized-controlled research design, assessing outcomes before and after the intervention. We hypothesized that, compared to a control group, participants in the intervention group would show a significant reduction in both self-reported and neural measures of error sensitivity from pre- to post-treatment. Additionally, we examined the potential long-term effects at an eight-week follow-up for self-report data. Objectives, hypotheses, methods, and pre–post differences in self-reported error sensitivity and the ERN were preregistered (https://osf.io/e528y). The follow-up assessment and the Pe analysis were exploratory and not preregistered.

## Method

### Participants

To investigate the potential efficacy of our online intervention, self-reported error sensitivity data were collected from 237 participants (*n* = 173 female, *n* = 61 male, and *n* = 3 divers), with a mean age of 24.35 years (*SD* = 7.04). From this sample, we also obtained EEG data for a subset of 69 participants (*n* = 45 female, *n* = 22 male, and *n* = 2 divers) with a mean age of 22.96 years (*SD* = 3.73). For the EEG analyses, an a priori sample size calculation was conducted using G*Power, version 3.1.9.7 (Faul et al., 2009; Faul et al., 2007), based on previous results stating a medium effect size of Cohen’s *d* = 0.46 (Meyer et al., 2020). A within-between interaction of group (intervention vs. control) × time (pre vs. post) of medium size (Cohen’s *f* > 0.23), indicating a group effect from pre to post measurement, can be detected with a sample size of *n* = 32 per group, a power of 95 %, and an alpha of 0.05 (Cohen, 1992). Therefore, we initially recruited 80 participants (*n* = 40 per group) ensuring a sufficient power for our EEG analyses even after the exclusion of participants due to drop out or low data quality.

### Procedure

Participants were recruited via internal student recruiting systems of the university, campus flyers and posters, as well as advertisements placed in social media and part-time jobs platforms for students. They were required to speak German fluently, to have normal or corrected-to-normal vision and no history of neurological disorders. All participants provided informed consent and received either course credit or monetary compensation for their participation. The study protocol was approved by the local ethics committee of the University of Hamburg (AZ: 2021_395) and was conducted in accordance with the Declaration of Helsinki (World Medical Association, 2013).

**Fig. 1:**
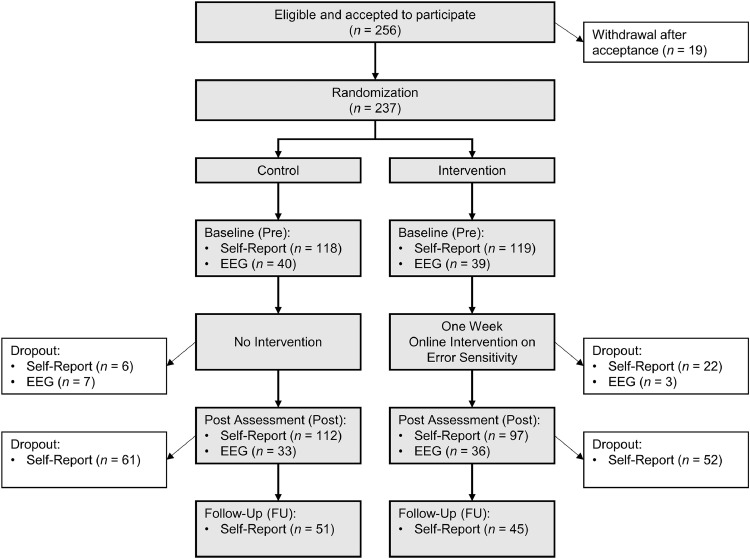
Overview of the study procedure including numbers of participants.

First, participants were randomly assigned to either the intervention group or the waitlist control group, with parallelization based on gender. This randomization procedure ensured a balanced design with groups of equal size. A battery of questionnaires was administered at baseline (Pre), post-intervention (Post), and follow-up (FU). The intervention group was instructed to complete the three consecutive sessions of the error sensitivity training online within one week, with a maximum of one session per day. As indicated by Fig. 1, drop-out rates at the post assessment differed significantly between the intervention and control groups: 22 participants in the intervention group (18.5%) compared to six participants in the control group (5.1%) discontinued their participation, χ^2^ (1) = 10.22, *p* = .001. This resulted in missing data at the post assessment for 28 participants (11.8%) of the total sample. There were no significant differences between participants with missing data and those with complete data in terms of gender, age, or self-reported baseline symptom severity on any measured outcome (all *p*s >.25).

The follow-up assessment was 58.38 days (*SD* = 14.93) on average after the post assessment. At follow-up, an overall of 74 participants in the intervention group (62.2%) compared to 67 participants in the control group (56.8%) lacked data, χ^2^ (1) = 0.72, *p* = .397, equaling a total of 141 participants (59.5%) with missing data at the follow-up assessment. This may be partly due to limited financial resources, as only course credit was available as compensation for participation at follow-up. There were no significant differences between participants with missing data and those with complete data in terms of gender, age, or self‑reported baseline symptom severity or pre–post change on any measured outcome (all *p*s > .10), with one exception: at follow‑up, participants with complete data were characterized by a greater pre-post reduction of self‑reported obsessive–compulsive symptoms than non‑completers, *t*(235) = -2.91, *p* = .004, *d* = -0.39.

Additionally, for the laboratory assessment, participants completed an arrowhead version of the flanker task at baseline (Pre) and after the intervention (Post). At the pre assessment, one participant of the intervention group (2.5 %) could not be included into the analysis due to less than six error segments needed to compute a reliable ERN (Olvet & Hajcak, 2009), while none of the control group had to be excluded, χ^2^ (1) = 1.01, *p* = .314. Missing data at the post-assessment due to fewer than six error segments or study discontinuation was observed in three participants in the intervention group (7.69 %) compared to seven participants in the control group (17.5 %), χ^2^ (1) = 1.72, *p* = .190.

### Intervention

Based on the previously published computerized intervention for reducing error sensitivity (Meyer et al., 2020), we developed an updated version tailored for German speaking adults. The aim of the intervention was to reduce error sensitivity by addressing participants’ evaluation of making errors, their emotional reactivity, and their subsequent behavior. Similar to the original English version, our intervention was not designed to target early or late stages of neural error processing specifically, as reflected by the ERN and Pe. Instead, it aimed to modulate overall sensitivity to errors. The program consisted of three sessions, each approximately 20 minutes, with participants required to complete all sessions within one week. It also included supplementary exercises between sessions to reinforce learning and facilitate transfer to everyday life, which is why participants were limited to completing only one session per day by default. The sessions were designed to build on each other, beginning with psychoeducation about error sensitivity (session 1), followed by exploring alternative behavioral responses to mistakes (session 2), and culminating in the formulation of a plan for future behaviors (session 3). Various methods were employed to convey the content effectively. Primarily, participants viewed slides accompanied by audio explanations providing additional detail. To enhance motivation and engagement, interactive elements such as memory games, short quizzes, drag-and-drop tasks, and input fields were incorporated. Additionally, participants were instructed to complete brief, guided homework exercises between sessions to facilitate the integration of the learned strategies into their daily routines.

Session 1 introduced the idea that mistakes are normal, useful for learning, and can be viewed constructively. As homework, participants were asked to reflect on situations in their daily lives where mistakes occur, how they personally react to and evaluate these errors, and how they perceive the mistakes made by others. Session 2 reviewed these insights, discussed participants’ homework experiences, and presented common behavioral reactions to errors, including avoidance and safety behaviors. Strategies for changing unhelpful responses were introduced. For homework, participants were asked to identify personal behaviors that may contribute to avoiding mistakes and developing plans to change them. Session 3 again reviewed earlier content and homework experiences. Participants then set a personal goal for how they want to handle errors and created a step-by-step plan to achieve it, including anticipating obstacles and solutions.

### Measures

Self-reported error sensitivity was assessed using the combined “Concern over Mistakes and Doubts” subscale (α_Pre_ = .90, α_Post_ = .90, α_FU_ = .90) of the Frost Multidimensional Perfectionism Scale (FMPS-D; 35 items, 5-point Likert scale 1-5; Frost et al., 1990; Stöber, 1998). For the German version, it is recommended to combine the two subscales “Concern over Mistakes” and “Doubts about Actions” (Stöber, 1998). We also assessed trait worry by the Penn State Worry Questionnaire (PSWQ; 16 items, 5-point Likert scale 1-5; α_Pre_ = .93, α_Post_ = .93, α_FU_ = .93; Glöckner-Rist & Rist, 2014; Meyer et al., 1990), obsessive-compulsive symptoms by the Obsessive-Compulsive Inventory Revised (OCI-R; 20 items, 5-point Likert scale 0-4; α_Pre_ = .91, α_Post_ = .93, α_FU_ = .91; Foa et al., 2002; Gönner et al., 2007), and depressive symptoms by the Beck Depression Inventory (BDI-II; 21 items, 4-point Likert scale 0-3, α_Pre_ = .93, α_Post_ = .94, α_FU_ = .94; Beck et al., 1996; Hautzinger et al., 2006). For the intervention group, we also assessed adherence to the training by asking participants at the end of the program to rate statements such as “During the online training … I paid attention” or “… I did the homework” (5 items, 4-point Likert scale 1-4, α = .73)

### Flanker Task

Sitting at a viewing distance of approx. 24 inches in front of a 19-inch monitor with a resolution 1920 × 1080 pixels and a refresh rate of 60 Hz, participants completed an arrowhead version of the flanker task (Eriksen & Eriksen, 1974) presented using Presentation Software (Neurobehavioral Systems, Inc., Albany, California). The stimulus set was five horizontally aligned arrows (one target and four flankers) which was approx. 6.2° in width and approx. 1.0° in height. The arrows pointed either in the same direction (<<<<< or >>>>>) or in opposite directions (<<><< or >><>>), with their orientation pseudo-randomly assigned. Participants were instructed to indicate the direction of the center arrow by pressing a key with their respective left or right index finger as quickly and accurately as possible. They practiced this with 20 trials that included immediate feedback on their performance. During the main task, no feedback was provided, deviating from the preregistered protocol.

The task consisted of five blocks of 80 trials each, for a total of 400 trials. Each trial had three phases: a fixation period lasting 200–1200 ms, presentation of the arrow stimuli for 100 ms, and a response window of up to 800 ms. Accuracy was calculated as the percentage of correct responses, response times as the interval between stimulus onset and the participant’s response, and post-error slowing (PES) was measured using a robust method; specifically, the average response time difference between the last correct trial before an error and the first correct trial after an error (Dutilh et al., 2012).

### Electrophysiological Recording and Processing

EEG signals were recorded using a cap equipped with 64 Ag/AgCl electrodes (Easycap, Herrsching, Germany) arranged according to the 10-10 system, and an actiCHamp Plus amplifier (Brain Products GmbH, Gilching, Germany). Signals were band-pass filtered from DC to 280 Hz and continuously digitized at a sampling rate of 1000 Hz. The reference electrode was placed at FCz, with the ground at Fpz. External electrodes were positioned at the left and right infraorbital sites to monitor vertical eye movements.

Data processing was performed using Brain Vision Analyzer, version 2.3 (Brain Products GmbH, Gilching, Germany). First, a band-pass filter with a low cutoff of 0.1 Hz, a high cutoff of 30 Hz (24 dB/oct roll-off), and a notch filter at 50 Hz were applied to the continuous EEG data. Ocular artifacts were then corrected using independent component analysis (ICA; Jung et al., 2000), with relevant components identified semi-automatically and manually verified through scalp topography distribution, component activation, and visual inspection of the corrected EEG signals.

Subsequently, the continuous data were re-referenced to the average of all scalp electrodes and segmented into response-locked epochs from -500 ms to 800 ms relative to the response. Segments containing artifacts (defined by an absolute voltage exceeding 200 µV, a voltage step greater than 50 µV between consecutive data points, or a maximum voltage difference of less than 0.5 µV within 100 ms intervals) were rejected. None of the participants were excluded from the analysis due to having more than 25% of their trials containing artifacts (Luck, 2014).

Finally, EEG data were corrected for the baseline interval of -500 ms to -300 ms (Klawohn, Meyer, et al., 2020; Sandre et al., 2020) and both ERN and CRN were quantified at FCz using mean amplitudes between 0 and 100 ms post response. Additionally, we also extracted mean amplitudes of the Pe and Pc at CPz in a time interval from 400 to 600 ms. The selection of electrode site was based on visual inspection of the maximal grand-averaged ERP signal. Spearman-Brown corrected correlations of odd- and even-numbered trials were used to investigate the psychometric properties for the ERN (*r*_Pre_ = .87, *r*_Post_ = .85), CRN (*r*_Pre_ = .99, *r*_Post_ = .98), Pe (*r*_Pre_ = .40, *r*_Post_ = .34), and Pc (*r*_Pre_ = .94, *r*_Post_ = .93).

### Data analysis

All statistical tests were performed using SPSS, version 29.0 (SPSS, Inc., Chicago), with a significance threshold of alpha = .05. Differences in gender between the intervention and control group were assessed using a χ^2^-test; differences in age, education years, and clinical variables between the groups were tested with independent samples *t*-tests, with group (intervention, control) as the between-subject factor.

To investigate our hypothesis regarding self-reported error-sensitivity (FMPS-CMD), an intent-to-treat analysis was conducted. The intention-to-treat analyses included all participants originally assigned to each group, regardless of whether they discontinued participation or had missing data at the post or follow-up assessments. Missing data were addressed using multiple imputation. The imputation model incorporated gender, age, group, and all questionnaire data from pre, post and follow-up assessments, with a total of 50 imputations performed. Changes in questionnaires scores (FMPS-CMD, PSWQ, OCI-R, and BDI-II) were assessed by 3 × 2 mixed-measures ANOVAs with time (Pre, Post, FU) as within-subject factor and group (intervention, control) as between-subject factor. The potential modulation of ERPs (ERN/CRN and Pe/Pc) by the intervention was analyzed using in per-protocol analyses including only participants with complete data sets at both pre and post assessments. Separate 2 × 2 × 2 mixed-measures ANOVAs including time (Pre, Post) and response (correct, incorrect) as within-subject factors as well as group (intervention, control) as between-subject factor were conducted. In general, for within-subject factors with more than two levels, the Greenhouse-Geisser correction was applied when the assumption of sphericity was violated. Additionally, follow-up analyses were conducted in cases of significant interactions, with post-hoc comparisons using Sidak-corrected *p*-values.

Explorative analyses on the influence of baseline questionnaire and ERP scores on the potential reduction have been conducted using separate multiple linear regression models of each post assessment questionnaire measure (FMPS-CMD, PSWQ, OCI-R, and BDI-II) and ERPs (ERN/CRN and Pe/Pc) incorporating the mean-centered score of the respective outcome at the pre assessment, group, and the interaction pre × group as predictors. To examine how adherence to the training influenced the interventions potential impact within the intervention group, we calculated residualized post-intervention outcome scores for both questionnaires (FMPS-CMD, PSWQ, OCI-R, and BDI-II) and ERPs (ERN/CRN and Pe/Pc) by controlling for baseline scores, rather than simply using pre-post difference scores. These residualized scores were then correlated with the adherence score using Pearson’s correlation coefficient.

## Results

### Full sample

#### Demographic and self-report data at baseline

As shown in Table 1, there were no differences between the groups in terms of gender, education, self-reported error sensitivity, or any clinical measures (all *p*s >.19). However, the intervention group was slightly younger (*M* = 23.36, *SD* = 5.58) than the control group (*M* = 25.34, *SD* = 8.16), *t*(235) = 2.18, *p* = .030, *d* = 0.28. Although this age difference of approximately two years was statistically significant, we do not consider it to be practically meaningful. However, we addressed the potential impact of age by conducting a sensitivity analysis in which age was included as a covariate.

**Table 1:** Demographical and self-report data across groups in the full sample.

	Control (*n* = 118)		Intervention (*n* = 119)		Group Comparison			
	*M*	*SD*	*M*	*SD*	*χ^2^ / t*	*df*	*p*	*d*
**Demographic**								
Gender (f/m/d)	85/31/2		88/30/1		0.40	2	.820	
Age (yrs)	25.34	8.16	23.36	5.58	2.18*	235	.030	0.28
Education (yrs)	12.36	0.61	12.26	0.69	1.22	235	.222	0.16
**Self-Report**								
FMPS-CMD	35.06	9.71	36.74	10.29	-1.29	235	.197	-0.17
PSWQ	50.36	12.00	51.50	11.63	-0.74	234	.458	-0.10
OCI-R	13.78	12.06	15.07	10.83	-0.86	234	.389	-0.11
BDI-II	9.19	10.03	8.94	7.43	0.21	234	.831	0.03

*Note.* Age and education in years; BDI-II = Beck Depression Inventory; FMPS-CMD = Frost Multidimensional Perfectionism Scale – Subscale Concern over Mistakes and Doubts; Gender (f = female, m = male, d = diverse/ non-binary); OCI-R = Obsessive-Compulsive Inventory Revised; PSWQ = Penn State Worry Questionnaire. * *p* < .05

#### Efficacy of the training on self-report data

**Fig. 2:**
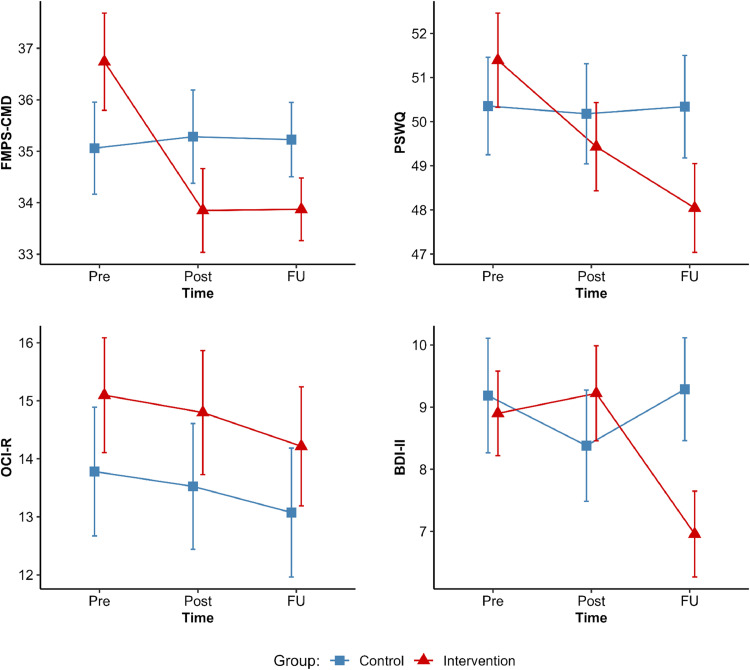
Results of the intent-to-treat analyses across time (Pre, Post, Follow-Up) for the control (*n* = 118) and intervention groups (*n* = 119). Error bars indicate standard error.

First, we investigated the efficacy of the one-week online training using intent-to-treat analyses across the pre-, post-, and follow-up assessments (Table 2 and Fig. 2). Results showed a successful reduction of self-reported error sensitivity (FMPS-CMD) by the intervention. This was indicated by the significant interaction effect of time × group, *F*(1.83, 429.52) = 11.41, *p* = <.001, $\eta_{p}^{2}$ = 0.05, ε = 0.91, which remained significant, even after entering age as covariate, *F*(1.83, 428.08) = 10.29, *p* = <.001, $\eta_{p}^{2}$ = 0.04, ε = 0.92. In the intervention group, FMPS-CMD scores decreased from pre to post assessment, *t*(118) = 5.13, *p* = <.001, *d* = 0.47 (completers: *t*(96) = 4.59, *p* = <.001, *d* = 0.47), and remained significantly lower at follow-up, *t*(118) = 4.19, *p* = <.001, *d* = 0.39 (completers: *t*(44) = 3.65, *p* = <.001, *d* = 0.54). In contrast, no changes were observed in the control group between pre and post assessment, *t*(117) = -0.52, *p* = .960, *d* = -0.05, nor between pre and follow-up assessment, *t*(117) = -0.33, *p* = .990, *d* = -0.03. Since the factor structure of the German version deviates from the original, we also analyzed our data using only the "Concern over Mistakes" subscale (FMPS-CM), which is identical to the original English version. The results showed an identical pattern, with a significant interaction effect of time × group, *F*(1.83, 429.82) = 8.53, *p* = <.001, $\eta_{p}^{2}$ = 0.04, ε = 0.92. Taken together, the intervention was found to be effective in reducing self-reported error sensitivity.

**Table 2:** Descriptive results of the intent-to-treat analyses across time (Pre, Post, Follow-Up) for the control (*n* = 118) and intervention groups (*n* = 119).

	Pre		Post		Follow-Up	
	*M*	*SD*	*M*	*SD*	*M*	*SD*
**FMPS-CMD**						
Control	35.06^a^	9.71	35.28^a^	9.84	35.23^a^	7.85
Intervention	36.74^a^	10.29	33.85^b^	8.89	33.87^b^	6.64
**PSWQ**						
Control	50.36^a^	12.00	50.18^a^	12.32	50.34^a^	12.63
Intervention	51.39^a^	11.64	49.43^b^	10.92	48.04^c^	10.98
**OCI-R**						
Control	13.78^a^	12.06	13.52^a^	11.77	13.07^a^	12.07
Intervention	15.10^a^	10.79	14.80^a^	11.65	14.22^a^	11.19
**BDI-II**						
Control	9.19^a^	10.03	8.38^a^	9.71	9.29^a^	9.00
Intervention	8.90^a^	7.42	9.22^a^	8.36	6.96^b^	7.54

*Note.* BDI-II = Beck Depression Inventory; FMPS-CMD = Frost Multidimensional Perfectionism Scale – Subscale Concern over Mistakes and Doubts; Gender (f = female, m = male, d = diverse/ non-binary); OCI-R = Obsessive-Compulsive Inventory Revised; PSWQ = Penn State Worry Questionnaire. Mean values with different subscripts within rows indicate significant differences according to Sidak corrected post-hoc *t*-tests with *p* < .05.

We also examined whether the reduction extended to other relevant outcomes (Table 2 and Fig. 2). Indeed, we found a similar time × group interaction for self-reported trait worry (PSWQ), *F*(1.88, 442.73) = 11.08, *p* = <.001, $\eta_{p}^{2}$ = 0.05, ε = 0.94, which was qualified by decreasing scores in the intervention group but not the control group. Specifically, in the intervention group, PSWQ scores decreased from pre to post assessment, *t*(118) = 3.58, *p* = <.001, *d* = 0.33, and were even lower at follow-up, *t*(118) = 5.68, *p* = <.001, *d* = 0.52. In contrast, trait worry remained on a constant level over time in the control group (all *p*s > .97). The training also influenced depressive symptoms (BDI-II), as evidenced by a significant time × group interaction, *F*(1.95, 458.39) = 10.43, *p* = <.001, $\eta_{p}^{2}$ = 0.04, ε = 0.98. In the control group, no significant changes were observed over time (all *p*s > .15). However, although there was no difference between pre- and post-assessment in the intervention group, *t*(118) = -0.63, *p* = .884, *d* = -0.06, a significant reduction was observed at follow-up compared to pre-assessment, *t*(118) = 3.74, *p* = <.001, *d* = 0.34. Finally, no impact of the training has been found for obsessive-compulsive symptoms (OCI-R), with the time × group interaction remaining insignificant, *F*(1.98, 464.08) = 0.48, *p* = .961, $\eta_{p}^{2}$ = 0.00, ε = 0.99.

**Fig. 3:**
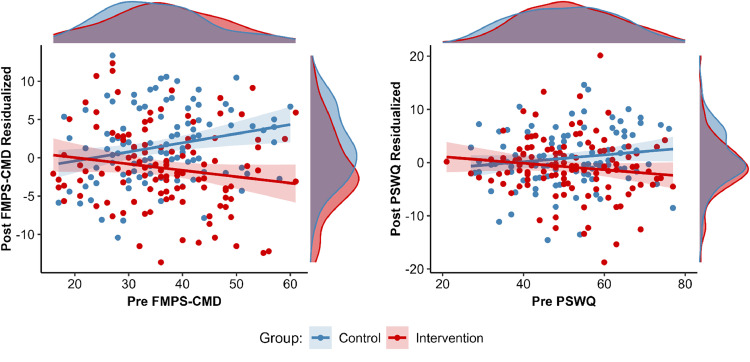
Scatter plot illustrating the association of baseline self-reported error sensitivity (FMPS-CMD, left panel) and trait worry (PSWQ, right) with reduction from pre- to post-intervention (positive values indicate increase, negative values indicate reduction).

**Fig. 4:**
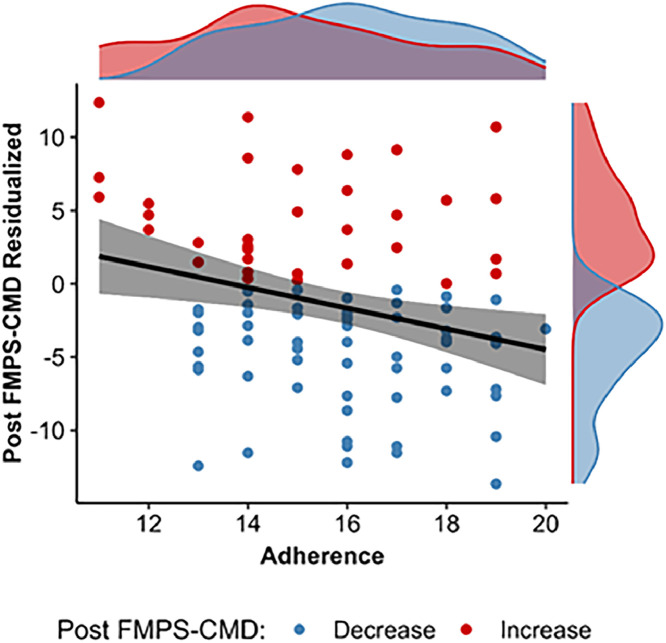
Scatter plot illustrating the association between training adherence and the change in self-reported error sensitivity (FMPS-CMD) from pre- to post-intervention.

In addition, we explored the influence of baseline scores on the potential reduction from pre to post assessment. A significant interaction of pre symptom level × group was found for the FMPS-CMD, *b* = -0.20, *SE* = 0.07, *p* = .003, and PSWQ, *b* = -0.13, *SE* = 0.05, *p* = .015, indicating that the reduction due to the intervention was greater in those with higher pre symptom level (Fig. 3). This result pattern was not found for the OCI-R and BDI-II (all *p*s >.21). Detailed results are provided in the supplementary materials (Table S1). Lastly, we examined how participants’ adherence to the training influenced the reduction in each self-reported outcome within the intervention group. Greater adherence significantly correlated with reductions in FMPS-CMD scores from pre- to post-intervention, *r* = -.28, *p* = .006 (Fig. 4). However, no significant correlations were found for the PSWQ, *r* = -.06, *p* = .563, OCI-R, *r* = .07, *p* = .496, or BDI-II, *r* = -.17, *p* = .104.

### EEG-subsample

#### Demographic and self-report data at baseline

The intervention and control group of the EEG subsample did not differ regarding gender, age, education, self-reported error sensitivity, or any clinical measure (all *p*s >.09). Details can be found in Table 3.

**Table 3:** Demographical and self-report data across groups in the EEG subsample.

	Control (*n* = 33)		Intervention (*n* = 36)		Group Comparison			
	*M*	*SD*	*M*	*SD*	*χ^2^ / t*	*df*	*p*	*d*
**Demographic**								
Gender (f/m/d)	20/11/2		25/11/0		2.43	2	.297	
Age (yrs)	23.45	3.73	22.50	3.72	1.06	67	.146	0.26
Education (yrs)	12.42	0.50	12.19	0.89	1.31	67	.098	0.32
**Self-Report**								
FMPS-CMD	32.18	8.98	35.00	10.74	-1.18	67	.122	-0.28
PSWQ	48.27	13.98	48.94	11.23	-0.22	67	.413	-0.05
OCI-R	12.15	11.89	11.31	7.97	0.35	67	.364	0.08
BDI-II	6.70	7.93	7.64	7.11	-0.52	67	.302	-0.13

*Note.* Age and education in years; BDI-II = Beck Depression Inventory; FMPS-CMD = Frost Multidimensional Perfectionism Scale – Subscale Concern over Mistakes and Doubts; Gender (f = female, m = male, d = diverse/ non-binary); OCI-R = Obsessive-Compulsive Inventory Revised; PSWQ = Penn State Worry Questionnaire. * *p* < .05

#### Efficacy of the training on error-related brain activity

As a manipulation check, we first examined whether the training also led to a reduction in self-reported error sensitivity (FMPS-CMD) from pre- to post-assessment within the EEG subsample. The results revealed a significant interaction of time × group, *F*(1, 67) = 4.25, *p* = .046, $\eta_{p}^{2}$ = 0.06, which was qualified by a reduction in the intervention group, *t*(35) = 2.11, *p* = .023, *d* = 0.35, but not in the control group, *t*(32) = -0.68, *p* = .556, *d* = -0.12.

**Fig. 5:**
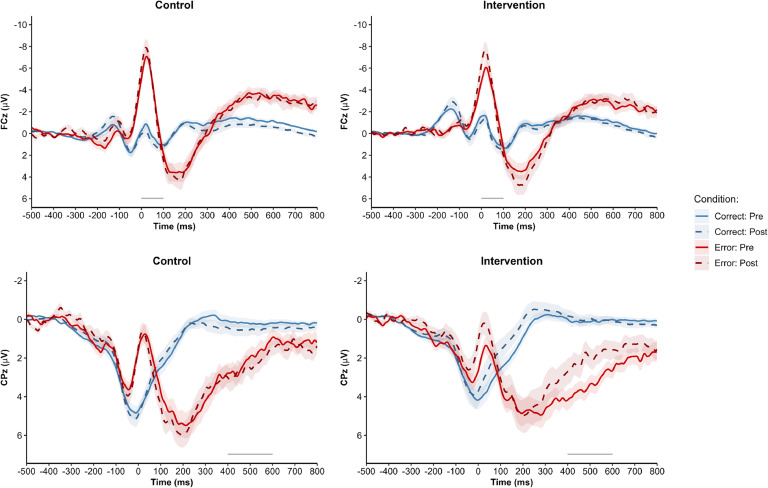
Response-locked grand-averaged waveforms of the EEG subsample across time (pre, post) for the control (*n* = 33) and intervention groups (*n* = 36) at electrode FCz (ERN, CRN) and CPz (Pe, Pc). Shaded error bars represent standard error. Scoring interval is depicted by a grey line at the bottom of each graph.

However, this reduction in self-reported error sensitivity following the intervention was not accompanied by a corresponding decrease in ERN or CRN amplitudes (Table 4 and Fig. 5). Neither the interaction of group × time, *F*(1, 67) = 0.13, *p* = .716, $\eta_{p}^{2}$ = 0.00, nor the interaction of group × time × response, *F*(1, 67) = 0.01, *p* = .910, $\eta_{p}^{2}$ = 0.00, approached significance. This indicates that neither the ERN nor the CRN changed over time in either group. In contrast, we identified a reduction in the Pe component (Table 4 and Fig. 5), as indicated by a significant of group × time × response interaction, *F*(1, 67) = 4.49, *p* = .038, $\eta_{p}^{2}$ = 0.06. This was driven by a reduction of the Pe in the intervention group, *t*(35) = 2.72, *p* = .004, *d* = 0.45, with no significant change in the control group, *t*(32) = -0.86, *p* = .460, *d* = -0.15. The Pc did not show any significant change over time in either group (all *p*s >.14). Overall, these results indicate that the intervention led to a reduction in the Pe component.

**Table 4:** Descriptive results of the EEG subsample across time (pre, post) for the control (*n* = 33) and intervention groups (*n* = 36).

	Pre		Post		Within Comparison			
	*M*	*SD*	*M*	*SD*	*t*	*df*	*p*	*d*
**Self-Report**								
**FMPS-CMD**								
Control	32.18	8.98	32.76	9.88	-0.68	32	.556	-0.12
Intervention	35.00	10.74	32.83	9.67	2.11*	35	.023	0.35
**ERPs**								
**ERN**								
Control	-3.73	3.43	-3.81	4.44	0.15	32	.886	0.03
Intervention	-3.45	4.61	-3.80	4.71	0.62	35	.525	0.10
**CRN**								
Control	0.16	2.16	0.67	2.25	-2.03	32	.275	-0.35
Intervention	-0.10	2.78	0.23	3.12	-0.58	35	.462	-0.10
**Pe**								
Control	2.06	2.61	2.42	2.79	-0.86	32	.460	-0.15
Intervention	3.38	2.48	2.00	2.98	2.72**	35	.004	0.45
**Pc**								
Control	0.20	1.31	0.53	1.61	-1.96	32	.148	-0.34
Intervention	0.10	1.32	0.00	1.50	0.36	35	.672	0.06
**Behavior**								
**Accuracy**								
Control	0.92	0.05	0.93	0.05	-2.28	32	.104	-0.40
Intervention	0.91	0.07	0.94	0.04	-3.02***	35	<.001	-0.50
**RT Error**								
Control	407.27	82.10	408.53	93.06	-0.19	32	.876	-0.03
Intervention	414.33	60.38	413.12	65.23	0.14	35	.876	0.02
**RT Correct**								
Control	452.72	50.85	437.60	45.30	4.91***	32	<.001	0.85
Intervention	467.76	38.37	450.00	35.85	4.25***	35	<.001	0.71
**PES**								
Control	37.86	27.84	28.02	29.19	1.93	32	.065	0.34
Intervention	56.52	26.68	27.81	23.32	5.59***	35	<.001	0.93

*Note.* CRN = correct-response negativity; ERN = error-related negativity; ERP = event-related potential; FMPS-CMD = Frost Multidimensional Perfectionism Scale – Subscale Concern over Mistakes and Doubts; Pc = correct positivity; Pe = error positivity; PES = post-error slowing; RT = response time. Post-hoc comparisons with Sidak corrected *p*-values. * *p* < .05, ** *p* < .01, *** *p* < .001

Similar to the self-reported data, we explored the influence of baseline magnitude and adherence on the potential ERP reduction from pre to post assessment. However, neither baseline ERP magnitude (all *p*s >.11), nor adherence were significantly associated with changes from pre to post assessment (all *p*s >.25). Detailed results can be found in the supplement (Table S2).

#### Efficacy of the training on behavioral performance

The training also enhanced behavioral performance (Table 4). Although the group × time interaction for accuracy was not statistically significant, *F*(1, 67) = 1.72, *p* = .195, $\eta_{p}^{2}$ = 0.03, within-group comparisons indicated higher accuracy in the intervention group from pre- to post-intervention, *t*(35) = -3.02, *p* = <.001, *d* = -0.50, whereas the control group showed no significant change, *t*(32) = -2.28, *p* = .104, *d* = -0.40. Regarding PES, a significant group × time × response interaction was observed, *F*(1, 67) = 6.76, *p* = .011, $\eta_{p}^{2}$ = 0.09, primarily driven by a reduction in PES within the intervention group, *t*(35) = 5.59, *p* = <.001, *d* = 0.93, but not in the control group, *t*(32) = 1.93, *p* = .065, *d* = 0.34. Finally, no significant differences were found between groups for response times; however, there was an overall reduction in response times on correct trials for both groups, *F*(1, 67) = 10.16, *p* = .002, $\eta_{p}^{2}$ = 0.13, indicating a general training effect of the flanker task. Similar to the ERP results, neither baseline behavioral performance (all *p*s >.08), nor adherence were significantly associated with changes from pre to post assessment (all *p*s >.50). Detailed results can be found in the supplement (Table S3).

## Discussion

This randomized-controlled study investigated the efficacy of a one-week online intervention designed to reduce both self-reported and neural error sensitivity in a sample of mainly university students. Intent-to-treat analyses indicated a medium-sized reduction of self-reported error sensitivity immediately after the intervention, which persisted over the period of eight weeks, as measured by the follow-up assessment. Similar results were observed for trait worry, but not for obsessive-compulsive symptoms. For depressive symptoms, the intervention did not produce an immediate effect, but a significant reduction was noted at follow-up. We also obtained EEG data from a subsample to examine the potential impact on neural signatures of error sensitivity. Although we expected a reduction in both early and late neural error processing (i.e., ERN and Pe), we observed differential effects: While the ERN remained unchanged, the Pe was reduced following the intervention. This reduction in the Pe implies diminished error significance and decreased allocation of cognitive resources to erroneous actions, suggesting that the intervention affects later evaluative processes, rather than early automatic error detection, as reflected by the ERN. This may be related to the fact that the intervention emphasizes cognitive reappraisal, psychoeducation, and behavioral planning, approaches mainly focusing on these evaluative, elaborative processes. Further effects on the earlier, more automatic neural error responses may require more time to be established. Additionally, the reported findings were accompanied by intervention-specific effects on behavioral performance, including increased accuracy and reduced post-error slowing. Moderation analysis revealed that higher baseline levels of self-reported error sensitivity and trait worry prior to the intervention were associated with greater reductions in the respective outcomes. Additionally, adherence to the intervention moderated the effect on self-reported error sensitivity, with higher adherence being associated with greater reductions indicating a dose–response relationship. No such moderation effect was observed for any of the other self-report, electrophysiological, or behavioral variables.

Consistent with the existing literature, we were able to replicate the efficacy of an error-specific intervention (Meyer et al., 2023; Meyer et al., 2020), particularly among participants with high self-reported error sensitivity and those who demonstrated a strong adherence to the intervention protocol. However, previous studies employed single-session designs with both pre- and post-assessments conducted during the same laboratory visit. These previous approaches make it difficult to distinguish between effects on trait versus state error sensitivity. In contrast, our study assessed the sustainability of the intervention’s effects by including a follow-up assessment eight weeks later. Replicating and extending the initial work of Meyer and colleagues, we demonstrated the reduction of self-reported error sensitivity in adults and that this effect even persisted for about eight weeks after the intervention. Furthermore, we found that the effects of the intervention generalized to other internalizing dimensions, including an immediate reduction in trait worry and a delayed positive effect on depressive symptoms at follow-up. These findings suggest that our intervention could serve as a powerful, easy-to-implement adjunct to traditional CBT, or as an early intervention strategy for individuals at risk for heightened error sensitivity and internalizing symptoms (Lahat et al., 2014; Meyer, 2017).

Regarding neural error sensitivity, our study yielded mixed findings. We found no evidence for a reduction in ERN amplitude, an effect previously observed in adults (Meyer et al., 2020) but not children (Meyer et al., 2023). Previous studies assessed the ERN within a few hours and a single session, potentially capturing rather state-related changes, whereas our intervention was designed to target more trait-like aspects of the ERN over a longer period. However, our intervention has not been found to affect ERN magnitude. This might be due to (i) insufficient intervention intensity, (ii) the trait-like stability of ERN, or (iii) the characteristics of our sample. Regarding intervention intensity, our one‑week intervention consisted of three sessions of approximately 20 minutes each. Given that the ERN is progressively shaped throughout childhood and adolescence by a range of biological and environmental factors and exhibits trait‑like characteristics in adulthood (Härpfer & Riesel, 2026), it is plausible that three brief sessions of a psychological intervention may not be sufficiently potent to sustainably alter the neural circuitry involved in the automatic detection of errors. Additionally, due to the trait‑like characteristics of the ERN, it may be particularly stable, potentially limiting its capacity for long‑term change per se. Another possible explanation for the absence of an ERN reduction could be that we recruited a non-clinical sample in which ERN amplitudes were likely within clinically irrelevant ranges. As a result, participants were not necessarily motivated to change their perspective on errors or their subsequent behavior, and many may not experience problematic levels of error sensitivity. Accordingly, the intervention may exert greater impact in populations characterized by a heightened ERN, such as individuals with anxiety disorders or OCD.

Instead of a reduction of the ERN, we observed a reduction in the Pe, a later error-related component that may reflect more elaborative and conscious error processing. Previous studies have already demonstrated the independence of early and late error processing, suggesting that ERN and Pe magnitude reflect dissociable processes (Di Gregorio et al., 2018; Nieuwenhuis et al., 2001; Overbeek et al., 2005). A reduced Pe might indicate a diminished tendency to engage in extended elaborative processing after errors, with errors being evaluated less significant, which could be beneficial in populations prone to excessive rumination or maladaptive post-error processing such as anxiety and OCD patients. The evaluation of a situation is an important factor in models of anxiety disorders and OCD, linking triggering situations to emotional reactivity and, consequently, behavior. For example, touching a door handle may be strongly associated with a fearful or disgusted emotional response or with compensatory behaviors such as excessive handwashing if the situation is evaluated as potentially dangerous, as in contamination-related fears. As such, the Pe might serve as a useful indicator of reappraisal and of how an individual has reevaluated their own actions throughout, for example, CBT treatment. Our findings might also suggest that interventions primarily focusing on cognitive reappraisal and error reevaluation, as in the present study, may be less effective in modifying the ERN, but may have a greater impact on the Pe. In fact, the highly automatic neural detection of errors, as reflected by the ERN, might be more responsive to interventions targeting automatic attentional tendencies. This is supported by studies utilizing attentional bias modification, which have demonstrated reductions of the ERN after administering single session trainings (Klawohn, Hajcak, et al., 2020; Nelson et al., 2015, 2017), as well as multiple sessions conducted over four to eight weeks (Amir et al., 2023; Tan et al., 2021).

A critical limitation that needs to be considered when interpreting our findings is the high dropout rate at the follow-up assessment. In fact, we were only able to obtain data from 40.5% of the original sample, likely because we were able to offer only course credit, rather than monetary compensation, at follow-up. As a result, a substantial portion of the data for our ITT analyses had to be estimated using a multiple imputation model, which potentially reduce the reliability of the observed effects. Although multiple imputation reduces bias under the assumption that data are missing at random, the high level of attrition still introduces uncertainty into the long‑term effect size estimates. Participants who did not complete the follow‑up may differ systematically from those who did, for example in engagement, symptom severity, or perceived benefit, which could influence the magnitude or stability of the observed outcomes. However, neither gender, age, nor self‑reported baseline symptom severity predicted discontinuation at follow‑up. Pre-post changes in self‑reported outcomes also did not predict discontinuation, with one exception: a greater reduction in self‑reported obsessive-compulsive symptoms was observed among participants who continued study participation at follow‑up, even though the intervention itself did not affect this outcome. Consequently, the generalizability of the eight‑week follow‑up findings should be interpreted with caution. Replication of these long‑term effects in future studies is needed, as the reductions observed at follow‑up may primarily reflect those who benefited most during the initial phase. Another limitation concerns the ERP results, where the split-half reliability of the Pe was low, ranging from .34 to .40. To address this, we excluded two participants with particularly low reliability, which increased the overall reliability of the Pe to .55 at both the pre- and post-assessments, while resulting in only minimal reductions in the statistical power of our analyses. When we repeated our mixed ANOVA for the Pe reduction, the interaction effect of interest still reached the significance threshold (*p* = .050).

In conclusion, our results demonstrate that the intervention represents a promising, accessible, and sustainably effective approach for individuals at risk of heightened error sensitivity and related internalizing symptoms. Although we did not observe a reduction of the ERN, the intervention's mitigating effect on the Pe may be particularly beneficial for individuals at risk for OCD, as an enhanced Pe has been discussed as a neural risk marker for this disorder (Bellato et al., 2021). Overall, neuroscientifically informed interventions hold considerable potential for contributing to the prevention and treatment of anxiety disorders and OCD by guiding the development of novel intervention approaches. In fact, individuals with heightened error sensitivity might benefit most from early interventions, potentially mitigating the progression or even preventing the subsequent development of an anxiety disorder or OCD. Additionally, combining traditional cognitive-behavioral approaches with mechanism-based trainings, such as an error-sensitivity training or attentional bias modification, may yield the most effective outcomes for reducing symptoms and lowering the risk of relapse. To this end, evaluating the efficacy of these interventions within clinical populations is essential.

## Declaration of Generative AI in Scientific Writing

During the preparation of this work the authors used ChatGPT (OpenAI, Inc.), version GPT 4.1 in order to improve the readability and language of the manuscript. After using this tool, the authors reviewed and edited the content as needed and take full responsibility for the content of the published article.

## Funding

This research was supported by the German Research Foundation (grant awarded to Anja Riesel DFG-Grant RI-2853/2-1) and the Open Access Publication Fund of the University of Hamburg (Project DEAL).

## CRediT authorship contribution statement

**Kai Härpfer:** Conceptualization, Data curation, Formal analysis, Investigation, Methodology, Project administration, Resources, Software, Visualization, Writing – original draft, Writing – review & editing. **Franziska M. Kausche:** Conceptualization, Investigation, Methodology, Project administration, Validation, Writing – review & editing. **Alexandria Meyer:** Conceptualization, Validation, Writing – review & editing. **Norman B. Schmidt:** Conceptualization, Validation, Writing – review & editing. **Anja Riesel:** Conceptualization, Funding acquisition, Methodology, Project administration, Resources, Supervision, Validation, Writing – review & editing.

## Declaration of competing interest

The authors declare the following financial interests/personal relationships which may be considered as potential competing interests:

Anja Riesel reports financial support was provided by German Research Foundation. Kai Härpfer reports article publishing charges was provided by Project DEAL. Kai Härpfer reports writing assistance was provided by OpenAI Inc. If there are other authors, they declare that they have no known competing financial interests or personal relationships that could have appeared to influence the work reported in this paper.

## Appendix Supplementary materials

Image, application 1
