# Supplementary material for: Targeting self-reported and neural error sensitivity: Short- and long-term effects of a one-week online intervention
Source: Int J Clin Health Psychol. 2026 Jan 23;26(1):100667. doi: 10.1016/j.ijchp.2026.100667 (PMC12861243; doi:10.1016/j.ijchp.2026.100667)
Supplement: Supplementary file 1 [file mmc1.docx]

**SUPPLEMENTARY MATERIALS**

**Tables**

**Table S1.** Multiple Regression Models Predicting Self-Report Data (Post-Assessment).

|  | *b* | *SE* | *p* | $R_{corr}^{2}$ | *F* | *df* | *p* |
| --- | --- | --- | --- | --- | --- | --- | --- |
| **Post FMPS-CMD** |  |  |  | .72 | 202.76*** | 3, 233 | <.001 |
| Pre FMPS-CMD | 0.90*** | 0.04 | <.001 |  |  |  |  |
| Group | -2.77*** | 0.63 | <.001 |  |  |  |  |
| Pre FMPS-CMD × Group | -0.20** | 0.07 | .003 |  |  |  |  |
| **Post PSWQ** |  |  |  | .79 | 291.89*** | 3, 233 | <.001 |
| Pre PSWQ | 0.93*** | 0.04 | <.001 |  |  |  |  |
| Group | -1.65* | 0.70 | .022 |  |  |  |  |
| Pre PSWQ × Group | -0.13* | 0.05 | .015 |  |  |  |  |
| **Post OCI-R** |  |  |  | .82 | 350.68*** | 3, 233 | <.001 |
| Pre OCI-R | 0.89*** | 0.05 | <.001 |  |  |  |  |
| Group | 0.05 | 0.62 | .932 |  |  |  |  |
| Pre OCI-R × Group | 0.08 | 0.07 | .215 |  |  |  |  |
| **Post BDI-II** |  |  |  | .67 | 160.61*** | 3, 233 | <.001 |
| Pre BDI-II | 0.84*** | 0.06 | <.001 |  |  |  |  |
| Group | 1.09 | 0.68 | .124 |  |  |  |  |
| Pre BDI-II × Group | 0.01 | 0.10 | .882 |  |  |  |  |

*Note.* BDI-II = Beck Depression Inventory; FMPS-CMD = Frost Multidimensional Perfectionism Scale – Subscale Concern over Mistakes and Doubts; OCI-R = Obsessive-Compulsive Inventory Revised; PSWQ = Penn State Worry Questionnaire. * *p* <.05, ** *p* <.01, *** *p* <.001

**Table S2.** Multiple Regression Models Predicting ERP Data (Post-Assessment).

|  | *b* | *SE* | *p* | $R_{corr}^{2}$ | *F* | *df* | *p* |
| --- | --- | --- | --- | --- | --- | --- | --- |
| **Post ERN** |  |  |  | .51 | 24.23*** | 3, 65 | <.001 |
| Pre ERN | 0.92*** | 0.16 | <.001 |  |  |  |  |
| Group | -0.22 | 0.77 | .765 |  |  |  |  |
| Pre ERN × Group | -0.16 | 0.20 | .391 |  |  |  |  |
| **Post CRN** |  |  |  | .25 | 8.64**** | 3, 65 | <.001 |
| Pre CRN | 0.82*** | 0.13 | <.001 |  |  |  |  |
| Group | -0.28 | 0.57 | .653 |  |  |  |  |
| Pre CRN × Group | -0.44 | 0.30 | .190 |  |  |  |  |
| **Post Pe** |  |  |  | .22 | 7.35*** | 3, 65 | <.001 |
| Pre Pe | 0.65*** | 0.17 | <.001 |  |  |  |  |
| Group | -1.16 | 0.61 | .060 |  |  |  |  |
| Pre Pe × Group | -0.18 | 0.23 | .439 |  |  |  |  |
| **Post Pc** |  |  |  | .40 | 15.96*** | 3, 65 | <.001 |
| Pre Pc | 0.98*** | 0.15 | <.001 |  |  |  |  |
| Group | -0.45 | 0.28 | .145 |  |  |  |  |
| Pre Pc × Group | -0.51 | 0.30 | .110 |  |  |  |  |

*Note.* CRN = correct-response negativity; ERN = error-related negativity; ERP = event-related potential; Pc = correct positivity; Pe = error positivity.* *p* <.05, ** *p* <.01, *** *p* <.001

**Table S3**. Multiple Regression Models Predicting Behavioral Performance (Post-Assessment).

|  | *b* | *SE* | *p* | $R_{corr}^{2}$ | *F* | *df* | *p* |
| --- | --- | --- | --- | --- | --- | --- | --- |
| **Post Accuracy** |  |  |  | .38 | 14.66*** | 3, 65 | <.001 |
| Pre Accuracy | 0.64*** | 0.14 | <.001 |  |  |  |  |
| Group | 0.01 | 0.01 | .167 |  |  |  |  |
| Pre Accuracy × Group | -0.36 | 0.20 | .093 |  |  |  |  |
| **Post RT Error** |  |  |  | .68 | 48.28*** | 3, 65 | <.001 |
| Pre RT Error | 1.03*** | 0.11 | <.001 |  |  |  |  |
| Group | -1.60 | 10.47 | .875 |  |  |  |  |
| Pre RT Error × Group | -0.33 | 0.19 | .085 |  |  |  |  |
| **Post RT Correct** |  |  |  | .76 | 73.19*** | 3, 65 | <.001 |
| Pre RT Correct | 0.84*** | 0.06 | <.001 |  |  |  |  |
| Group | 12.39 | 14.54 | .378 |  |  |  |  |
| Pre RT Correct × Group | -0.11 | 0.13 | .374 |  |  |  |  |
| **Post PES** |  |  |  | .12 | 4.02* | 3, 65 | .011 |
| Pre PES | 0.50*** | 0.14 | <.001 |  |  |  |  |
| Group | -6.95 | 6.26 | .258 |  |  |  |  |
| Pre PES × Group | -0.28 | 0.18 | .120 |  |  |  |  |

*Note.* PES = post-error slowing; RT = response time. * *p* <.05, ** *p* <.01, *** *p* <.001
